# Variations in rhizosphere soil total phosphorus and bioavailable phosphorus with respect to the stand age in *Pinus massoniana* Lamb.

**DOI:** 10.3389/fpls.2022.939683

**Published:** 2022-08-01

**Authors:** Yaowen Xu, Xiaogai Ge, Benzhi Zhou, Lei Lei, Wenfa Xiao

**Affiliations:** ^1^Research Institute of Subtropical Forestry, Chinese Academy of Forestry, Hangzhou, China; ^2^Qianjiangyuan Forest Ecosystem Research Station, National Forestry and Grassland Administration of China, Hangzhou, China; ^3^State Forestry Administration, Key Laboratory of Forest Ecology and Environment, Research Institute of Forest Ecology, Environment and National Protection, Chinese Academy of Forestry, Beijing, China

**Keywords:** phosphorus fractions, bioavailable phosphorus, phosphorus recycling, rhizosphere soil, stand age

## Abstract

Phosphorus (P) is a nutrient limiting plant growth in subtropical regions. However, our understanding of how soil P responds to an increase in stand age is rather poor. In particular, little is known about how bioavailable P pools (soluble P, exchangeable P, hydrolyzable P, and ligand P) shift with a change in stand age. Moreover, the P cycle in rhizosphere soil has the most direct and significant influence on plants. The aim of the present study was to determine the concentrations of total P in various rhizosphere soil bioavailable P fractions in 5-, 9-, 19-, 29-, and 35-year-old stands of *Pinus massoniana* Lamb. According to the results, total P (TP) concentration and N:P ratio in rhizosphere soil first decreased, and then increased with an increase in stand age. Soluble P concentration decreased first, and then increased with an increase in stand age; exchangeable P and ligand P decreased first, and then tended to be stable with an increase in stand age, whereas hydrolyzable P increased first, and then decreased. Structural Equation Model results suggested that ligand P and soluble P were the major factor affecting the TP. In addition, soil microorganisms and acid phosphatase-driven hydrolyzable P play a crucial role in soil bioavailable P cycling. Overall, the results of our study provide a mechanistic understanding of soil bioavailable P cycling under low available P conditions, and a basis for an effective P management strategy for the sustainable development of *P. massoniana* plantations.

## Introduction

Phosphorus (P) is one of the factors limiting tree productivity the most in tropical and subtropical ecosystems, especially in forests (Wright et al., 2018; Hou et al., 2020). Unlike carbon (C) and nitrogen (N), which enter the soil through biological fixation (photosynthesis and biological N fixation), P is basically derived from weathering of primary phosphate mineral sources (Gao et al., 2019). Once deposited in soil, P is converted into multiple co-existing inorganic and organic forms through complex chemical and biological processes (Chen et al., 2020). In addition, iron (Fe) and aluminum (Al) oxides can convert soluble P into a stable form that biota cannot exploit (Maranguit et al., 2017; Wang et al., 2017). Therefore, plants can adopt some strategies to cope with P deficiency and increase P supply, including biological processes (root exudates) that promote P supply. The balance between plant P uptake and soil P supply changes with stand age. However, little is known about the dynamics and mechanisms of change in P bioavailability with change in stand age.

P deficiency is a widespread phenomenon in subtropical forest ecosystems (Wu et al., 2011). The soil P cycle in forest ecosystems depends mainly on the internal system cycle. With the increase in forest age, the demand for P by trees, the composition and structure of forest ecosystems, and the distribution pattern and circulation law of soil P are affected by the P demand of trees (Wu et al., 2020). In addition, soil P exists in a variety of forms, including soluble inorganic P, insoluble inorganic P, and surface-adsorbed P (Bieleski, 1973). Transformation between different P fractions is regulated jointly by plants and microorganisms. For example, the demand for P in plants promotes the secretion of organic acids in plant fine roots, thus transforming insoluble organic P and inorganic P into soluble inorganic P (Fan et al., 2020). Recent research has highlighted that the P cycle in soils is strongly controlled by soil microbial biomass (Spohn and Widdig, 2017). In soils, microorganisms account for 68–78% of total P in the biomass and are involved in the accumulation and conversion of P in different soil P components (Turner et al., 2013). Data from previous studies suggest that soil microorganisms accelerate the release of unstable P by secreting hydrolases such as acid phosphatase, thereby breaking down organic P (Chen et al., 2018). In addition, when there is a cation–anion imbalance in a microorganism, it releases H^+^ or OH^−^ to maintain charge balance between the inside and outside of the cell, thus altering soil pH and regulating P bioavailability (Deluca et al., 2015). In alkaline soil, microorganisms release H^+^, which reduces the solubility of Ca ions or Ca oxides, and further reduces the degree of binding of Ca compounds to P ions, in turn releasing phosphate ions available to plants and increasing P availability (Devau et al., 2009; Deluca et al., 2015). However, in acidic soil, microorganisms release OH^−^, which reduces Fe and Al oxide activity, reduces the coupling of metal ions to phosphoric acid particles, and releases the coupled phosphoric acid ions into the soil solution, thus enhancing soil P availability (Devau et al., 2009; Deluca et al., 2015). In addition, considering the complex interactions between plants, microbes, and soil, several chemical conditions of rhizosphere soil are distinct from those of non-rhizosphere, which directly affect plant growth, development, and the absorption and use of water and nutrients. Furthermore, beneficial and harmful organisms survive and reproduce in the soil, modifying the response of plants to adverse situations (Jian et al., 2022). Regrettably, most of the current studies on the soil P cycle focus on non-rhizosphere soils, although it is the P cycle in rhizosphere soils that has the greatest and the most direct impact on plants (Wu et al., 2019a,b; Yang et al., 2021). Therefore, it is necessary to explore the strategies of P absorption and utilization in artificial forests from the perspective of P morphological characteristics in rhizosphere soils of different stand ages.

There is no unified standard for determining soil P fractions, although the methods of Chang and Jackson (1957) and Hedley et al. (1982) are widely adopted in the study of soil P fractions. For instance, using the methods proposed by Hedley et al. (1982) and Wang et al. studied the variations in soil P fractions in ginger fields (Wang et al., 2022). Using the methods proposed by Chang and Jackson (1957) and Adetunji (1994) studied the P requirements of corn–cowpea plantations. These methods have proven to be very useful in studies related to agriculture, as they provide direct indicators of P fertility; however, these methods do not adequately reflect rhizosphere processes (Johnson et al., 2003). P solubilized by rhizosphere processes, particularly by organic acids and extracellular enzymes, is not represented in these methods (Deluca et al., 2015). Furthermore, according to the different activities of each component of soil P, such fractionations dynamics may be well correlated with P accumulation and soil development, but they do not provide insight into the underlying mechanisms of P uptake or rhizosphere P conversion that drive ecosystem P dynamics (Deluca et al., 2015). Therefore, in order to better understand the mechanism of P utilization in plants, we need to use a targeted fractionation method, which can reflect the biologically mediated changes in available P and is sensitive to landscape-scale changes in soil P status.

As a unique native tree species in China, *Pinus massoniana* Lamb. has become the main pioneer tree species used in subtropical barren mountain afforestation because of its strong adaptability and drought resistance, with an estimated coverage area of 5.7 million hectares (Zhang et al., 2013). Research has shown that the soil total P (TP; 0.33 g·kg^−1^/4.04 × 102 g·m^3^) and available P storage (1.63 mg·kg^−1^/2.05 g·m^3^) of *P. massoniana* plantations were far lower than the average soil TP (8.2 × 102 g·m^3^) and available P storage (4.8 g·m^3^) of tropical and subtropical forests in China (Zhang et al., 2005). The low availability of P in the soil is one of the most important factors leading to the reduced productivity in *P. massoniana* plantations. However, little is known about the mechanism of the changes in soil bioavailable P in *P. massoniana* plantations.

In the present study, we selected *P. massoniana* plantations across different age classes to analyze changes in rhizosphere soil P fraction concentrations, physical and chemical properties, and microorganisms. We used the bioavailable-based P (BBP) method to determine the soil bioavailable P fractions; this is a novel method based on biological P utilization in the rhizosphere process and has not been reported in previous studies (Deluca et al., 2015; Gao et al., 2019). First, because of absorption by plants and nutrient return, P in the *P. massoniana* forest ecosystem shifts from acquiring system to recycling system; consequently, we hypothesized that (i) TP concentration in rhizosphere soil first decreases and then increases with an increase in stand age. Second, because different P fraction can be converted to each other, we hypothesized that (ii) change in soil TP concentration is caused by changes in one or more types of soil bioavailable P. Third, subtropical forest soil is rich in soil microbes; therefore, (iii) changes in soil bioavailable P concentrations are correlated with microbial community composition and enzyme activity. The results of the present study could facilitate the formulation of effective P management strategies and offer a theoretical basis for the sustainable development of *P. massoniana* plantations.

## Materials and methods

### Study site description

**Figure 1:**
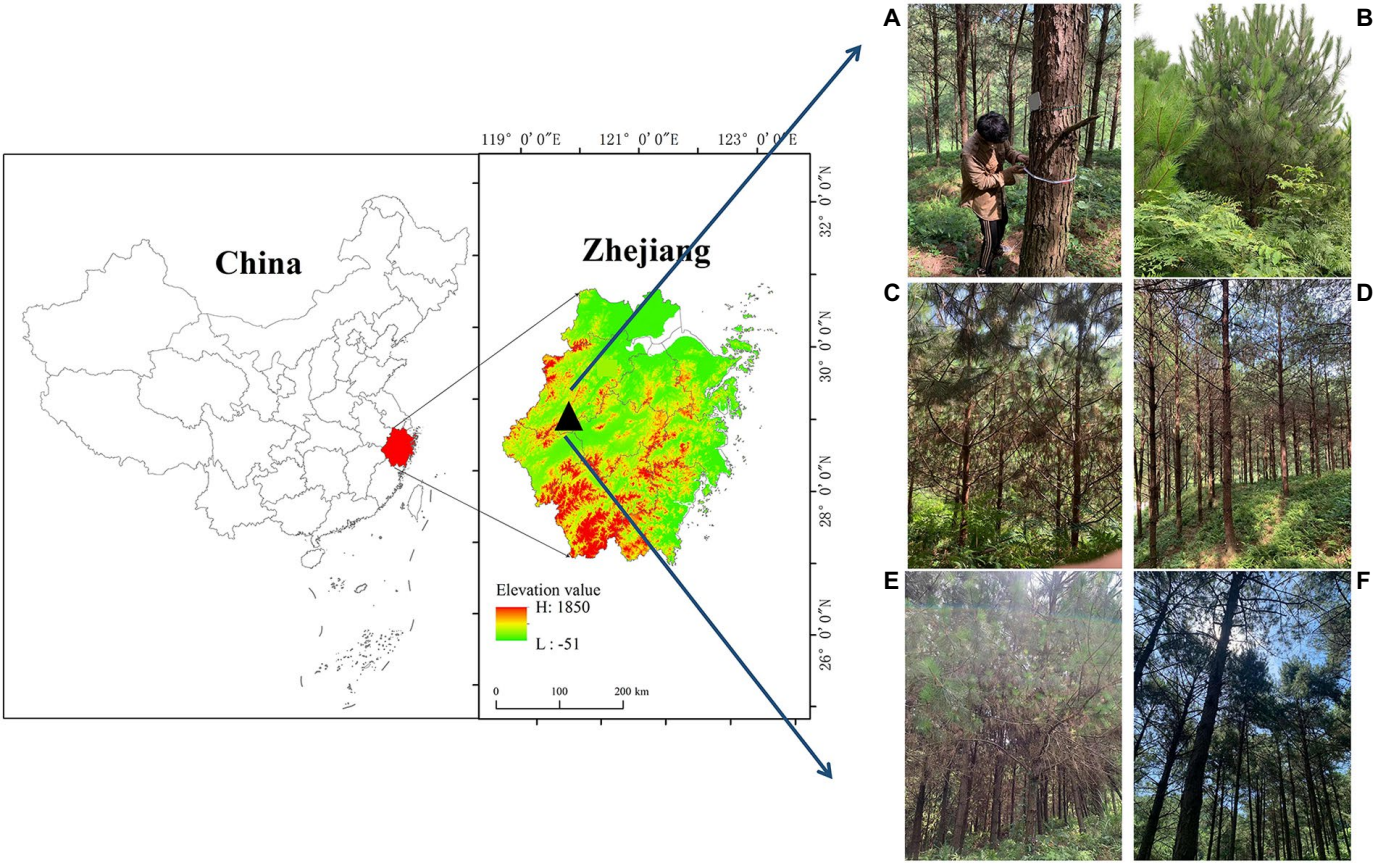
The study area is located in the Laoshan Forest Farm, Zhejiang Province, China. Tree survey **(A)**, 5-year-old plantations **(B)**, 9-year-old plantations **(C)**, 19-year-old plantations **(D)**, 29-year-old plantations **(E)**, and 35-year-old-plantations **(F)**.

This study was conducted at the Laoshan Forest Farm (29° 33′ N, 119° 03′ E; altitude, 152 m above sea level) in the Zhejiang Province, China, which has a subtropical monsoon climate (Figure 1). According to data recorded at a meteorological observation station about 7 km from the study area, the mean annual air temperature between 1980 and 2019 was 17.58°C, and the annual precipitation is 1,350 mm. The study area has two types of soil: red soil and lithological soil. The soil thickness is generally 30–120 cm, and the topsoil layer is 15–30 cm (Zhang et al., 2021).

### Experimental design

Due to the rapid growth in wood demand over the 20th century, China established several tree breeding bases, including our study sites (National *P. massoniana* improved seed base of Laoshan Forest Farm). The plantations in the study area have been established since the 1970s. Workers have harvested native trees and burned them on site, and then planted *P. massoniana* clones. In the first 3 years after planting, the workers cut down the undergrowth shrubs annually to promote seedling growth. Afterward, no management activities are carried out. Before the establishment of the plantation in the present study, the site conditions were as uniform as possible. The soil is mainly red soil and lithologic soil, with thin soil layer and poor nutritional status, and understory shrubs of the *P. massoniana* plantations were composed of *Rosa multiflora*, *Smilax china*, etc.

Field surveys and sampling were conducted in August 2021. *P. massoniana* plantations established in 1986, 1993, 2002, 2012, and 2016 were selected using the space-for-time (chronosequence) method (Figure 1). Although the chronological approach may be inadequate because of the difficulty in avoiding differences between plots, careful site selection and consideration of developmental linkages can enhance the reliability of chronological studies (Walker et al., 2010). The sample plots selected for this study were all at similar elevations. Three 20 m × 20 m quadrats were randomly set for each stand age of *P. massoniana* plantation, and the distance between each quadrat was approximately 30 m.

### Soil sample collection

Five standard trees of each were selected at random in each quadrat for rhizosphere soil samplings and then fully mixed these sampling points as a representative rhizosphere soil sample. The diameter at breast height of the standard tree was the average diameter at breast height for all trees in the quadrat, and there were no diseases and pests. Specifically, about a meter from each standard tree, soil was dug from a depth of 0–20-cm and the soil tightly attached to the surface of the fine roots gently shaken. Subsequently, the soil attached to the fine roots was brushed off gently and collected, and was regarded as the rhizosphere soil (Cui et al., 2019). The collected rhizosphere soil was divided into two parts: one part was dried naturally for the determination of total N (TN) and TP, and the other part was stored at 4°C for the determination of bioavailable P, microbial community composition, acid phosphatase, and phytase activity.

### Chemical analysis of soil samples

The rhizosphere soil pH was determined using a 1:2.5 soil:water solution (w/v), the TP concentration was analyzed using the ammonium molybdate method with colorimetry, and the TN concentration was measured using an elemental analyzer (CHN-O-RAPID, Heraeus, Germany; Ge et al., 2020). Phospholipid fatty acid (PLFA) analysis of the soil microbial community composition was performed using a gas chromatograph (Agilent Technologies 7890, Wilmington, DE, United States). The quantity (nmol·g^−1^ dry soil) of each PLFA was calculated based on the 19:0 internal standard (5 μg·ml^−1^), and the concentration of PLFA was used to estimate the biomass of specific microbiota in the soil sample. Fungi (F) include 18:2ω6c and 18:1ω9c; *Actinomyces* (ACT) include 10Me16:0, 10Me 17:0, and 10Me 18:0; arbuscular mycorrhizal fungi (AMF) include 16:1ω5c; Gram- (G-) include 16:1ω7c, 16:1ω9c, 18:1ω7c, 18:1ω5c, cy17:0, and cy19:0; Gram+ (G+) include i14:0, i15:0, a15:0, i16:0, a16:0, and i17:0; and bacteria (B) include i14:0, i15:0, a15:0, i16:0, a16:0, i17:0, 16:1ω7c, 16:1ω9c, 18:1ω7c, 18:1ω5c, cy17:0, cy19:0, 14:00, 15:00, 16:00, 17:00, and 16:1 2OH (Malik et al., 2008; Wang et al., 2020). The enzyme substrate fluorescence microplate method was used to determine related enzyme activities: Acid phosphatase (AP) activity was oxidized by 1 nmol p-nitroaniline per gram of soil per minute as an enzyme activity unit, and the phytase (PE) activity was determined using 1 μmol of inorganic p released from 5 mmol L^−1^ phytate sodium solution per gram of soil sample per hour at a pH of 5.5 (Saiya-Cork et al., 2002).

**Figure 2:**
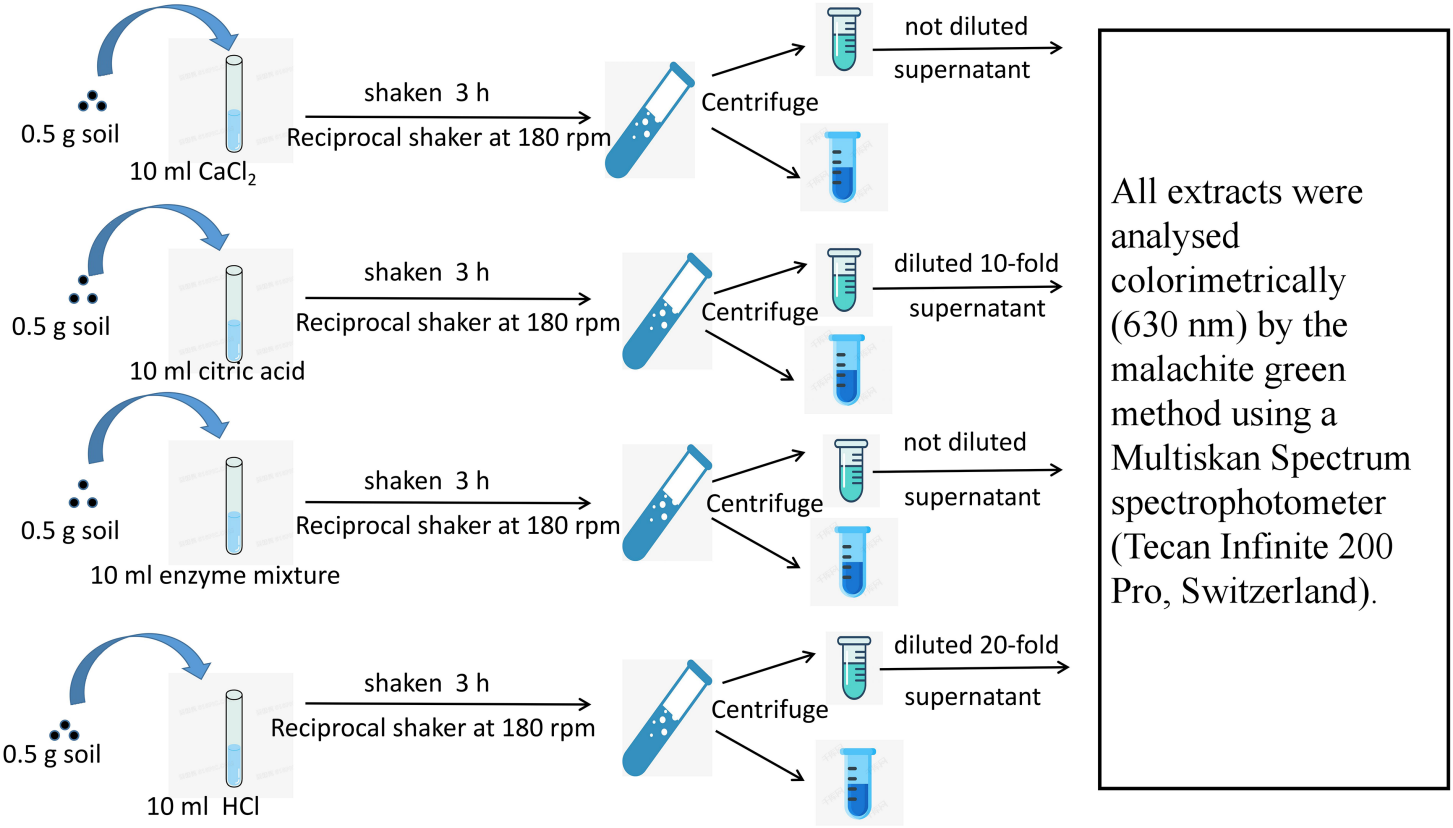
Fraction sequential extraction methods for the four types of soil bioavailable P.

Four soil bioavailable P pools were determined: (1) soluble P, which is readily absorbed by plants in small amounts and directly absorbed by root hairs and arbuscular mycorrhizas from soil solutions; (2) exchangeable P, which is a type of active P that is adsorbed on the surface of clay particles; when plants secrete organic acids, P in this form can be released into the soil solution; (3) hydrolyzable P, which is a type of active organic P that can be mineralized by AP and PE; and (4) ligand P, which is difficult to utilize by plants and can only be released into the soil solution by proton replacement or physical shock secreted by plant roots and microorganisms. Soluble P was extracted with 10 mM CaCl_2_; exchangeable P was extracted with 10 mM citric acid solution; hydrolyzable P was extracted with 0.02 U ml^−1^ phytase and acid phosphatase solution; and ligand P was extracted with 10 mM HCl, and the specific process is shown in Figure 2 (Deluca et al., 2015).

### Statistical analysis

One-way analysis of variance (ANOVA) was used to compare the effects of different stand ages on the TP, N:P ratio, bioavailable P, AP activity, and PE activity of rhizosphere soil. Analyses were performed using SPSS (version 23.0; IBM, Armonk, NY, United States), and the significance level of all statistical tests was set at *p* < 0.05. The redundancy analysis (RDA) was used to evaluate the relationship between the microbial community composition, enzyme activity, and four types of soil bioavailable P. AMOS 25.0 (SPSS Inc., Armonk, NY, United States) was used for path analysis of the four types of soil bioavailable P and TP.

## Results

### Rhizosphere soil TP concentration and four types of soil bioavailable P

**Figure 3:**
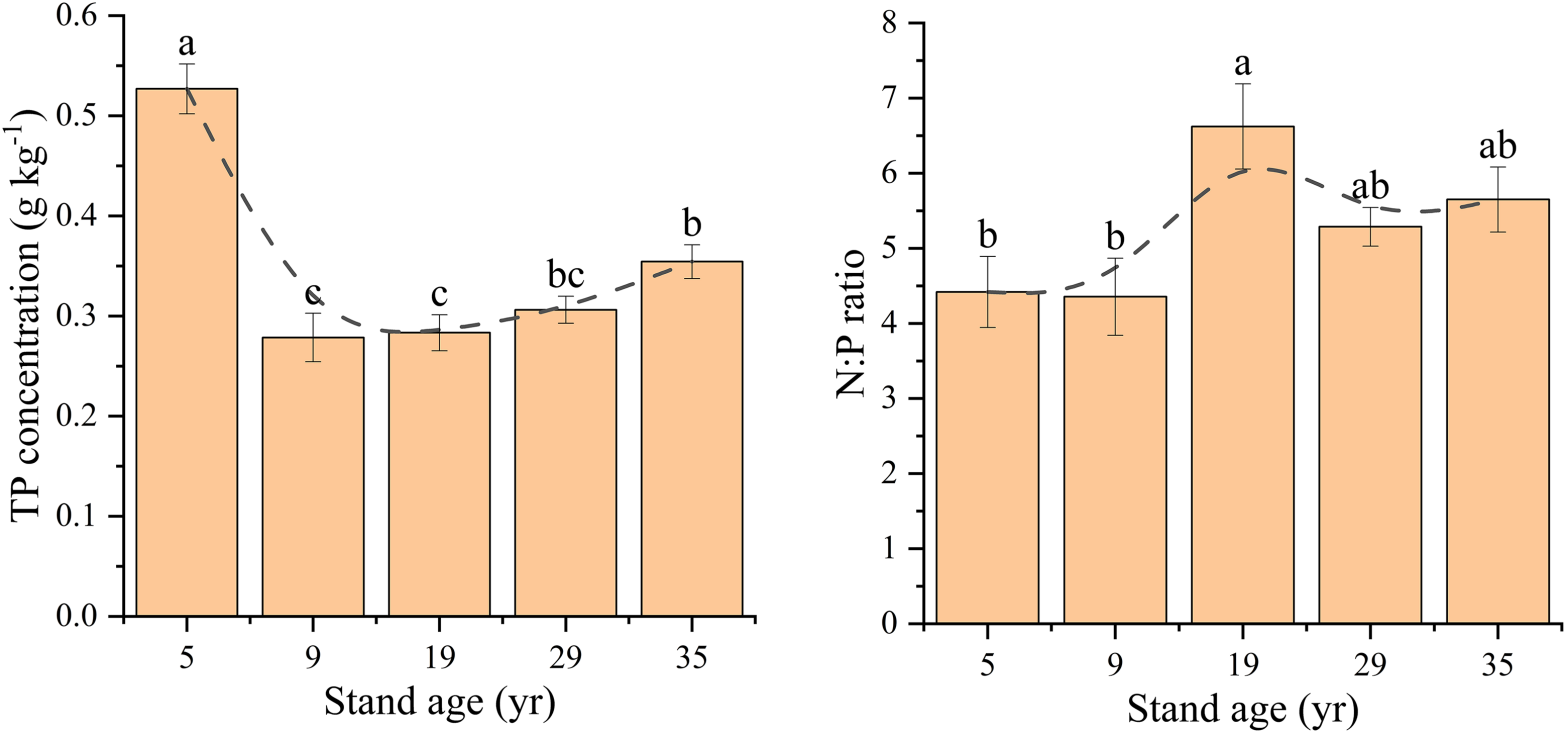
Changes in the total P concentrations and N:P ratio in the rhizosphere soil depending on stand age. Different lowercase letters indicate significant difference at the 0.05 (*p* < 0.05) level among the different stand ages.

The TP concentration in the rhizosphere soil first decreased, and then increased with increasing stand age (Figure 3). The TP concentration in the 5 years rhizosphere soil was 0.53 g kg^−1^, which was significantly higher than that in the other four stand ages (*p* < 0.05). The TP concentration was the lowest in the rhizosphere soil at 9 years, which was 0.28 g kg^−1^, and increased gradually with the increase in stand age until it reached 0.35 g kg^−1^ at 35 years. The N:P ratio in the rhizosphere soil ranged from 4.36 to 6.62 in the five stand ages, which increased first, and then decreased with the increase in stand age, reaching a maximum of 6.62 in 19 years.

**Figure 4:**
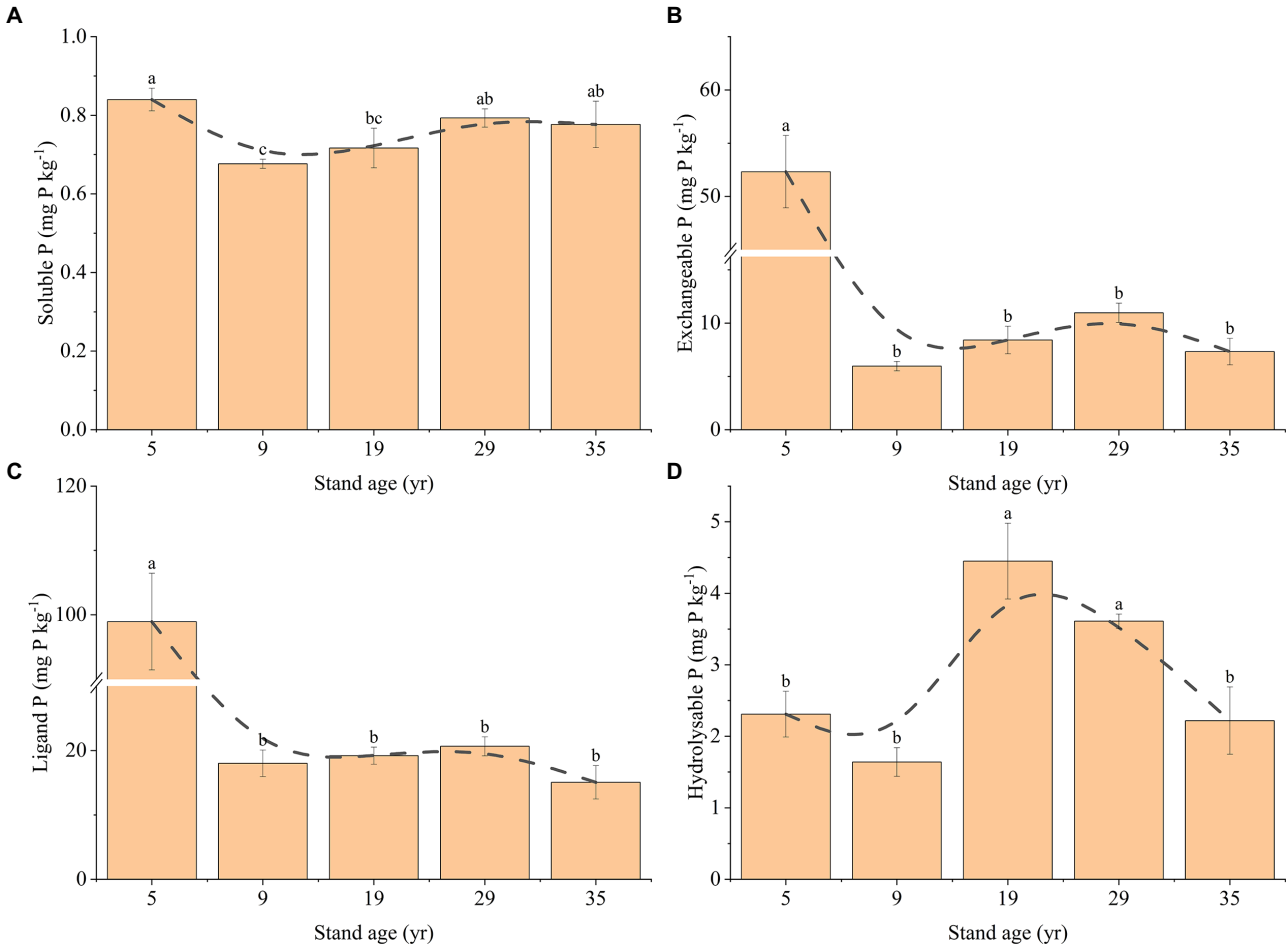
Changes in the concentrations of soluble P **(A)**, exchangeable P **(B)**, ligand P **(C)**, and hydrolyzable P **(D)** in the rhizosphere soil, depending on the stand ages. Different lowercase letters indicate a significant difference at the 0.05 (*p* < 0.05) level among the different stand ages.

Stand age was observed to have a significant effect on the rhizosphere soil bioavailable P (Figure 4). The soluble P concentration decreased first, and then increased with the increase in stand age, reaching the lowest value of 0.68 mg P kg^−1^ at the 9-year-old stand age and gradually increasing to 0.78 at the 35-year-old stand age (Figure 4A). The exchangeable P concentration at 5 years was 52.33 mg P kg^−1^, which was significantly higher than that of the other stand ages (Figure 4B). The hydrolyzable P concentration first increased, and then decreased with increasing stand age, reaching the highest value of 4.45 mg P kg^−1^ at 19 years and gradually decreasing to 2.22 mg P kg^−1^ at 35 years (Figure 4C). The ligand P concentration at the age of 5 years (98.95 mg P kg^−1^) was significantly higher than that of the other stand ages (*p* < 0.05; Figure 4D). There was no significant difference in the ligand P concentration between 9, 19, 29, and 35 years (*p* > 0.05).

**Figure 5:**
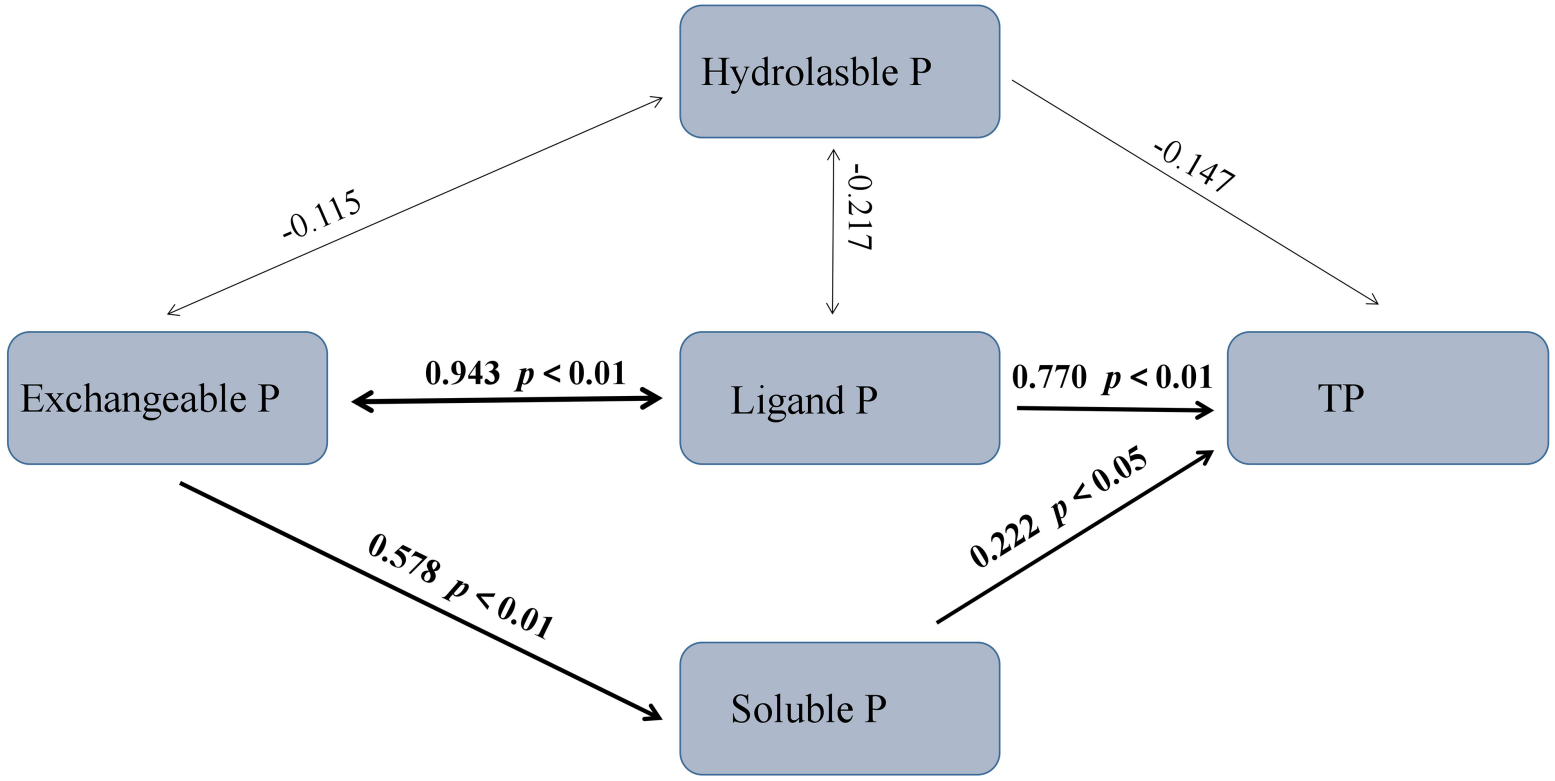
Structural equation model (SEM) showing the transformation between different rhizosphere soil P fractions. Numbers on arrows are standardized path coefficients. Arrow thickness represents the magnitude of the path coefficient. The overall fit of the model: *χ*^2^ = 0.730, *p* = 0.866, comparative fit index (CFI) = 1.000, RMR = 0.011.

According to the Structural Equation Model (SEM), the ligand P and soluble P were the major factors affecting TP, with path coefficients of 0.770 and 0.222, respectively (Figure 5). The exchangeable P fraction directly affected the soluble P fraction, with a path coefficient of 0.578. The exchangeable P fraction interacted with the ligand P fraction, and there was a positive covariance relationship (path coefficient = 0.943).

### AP, PE, and microbial community composition in the rhizosphere soil

**Figure 6:**
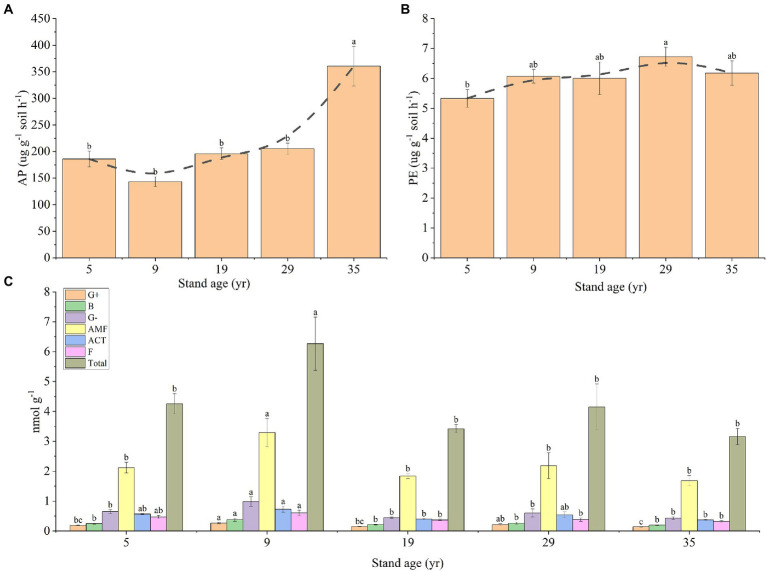
Changes in the concentrations of AP **(A)** and PE **(B)** in the rhizosphere soil, depending on the stand ages, AP, PE, and microbial communities in the rhizosphere soil. Effects of stand age on the PLFAs of microbial communities in the rhizosphere soil **(C)**. Different lowercase letters indicate a significant difference at the 0.05 (*p* < 0.05) level among the different stand ages.

The concentration of AP in the rhizosphere soil at 35 years was significantly higher than that at the other stand ages (*p* < 0.05), while the concentration of AP in the rhizosphere soil of the other four stand ages had no significant difference (*P* > 0.05), ranging from 143.19 to 185.99 μg g^−1^ soil h^−1^ (Figure 6A). The PE concentration in the rhizosphere soil first increased, and then decreased with the increase in stand age, and the highest value was 6.73 μg g^−1^ soil h^−1^ (29 years; Figure 6B). The PLFAs concentration of the G+,G−, B, AMF, ACT, and F communities and the total PLFAs concentrations were higher than that of other forest ages at 9 years (*p* < 0.05; Figure 6C).

### Relationships between rhizosphere soil P fractions and rhizosphere soil biochemical properties

**Figure 7:**
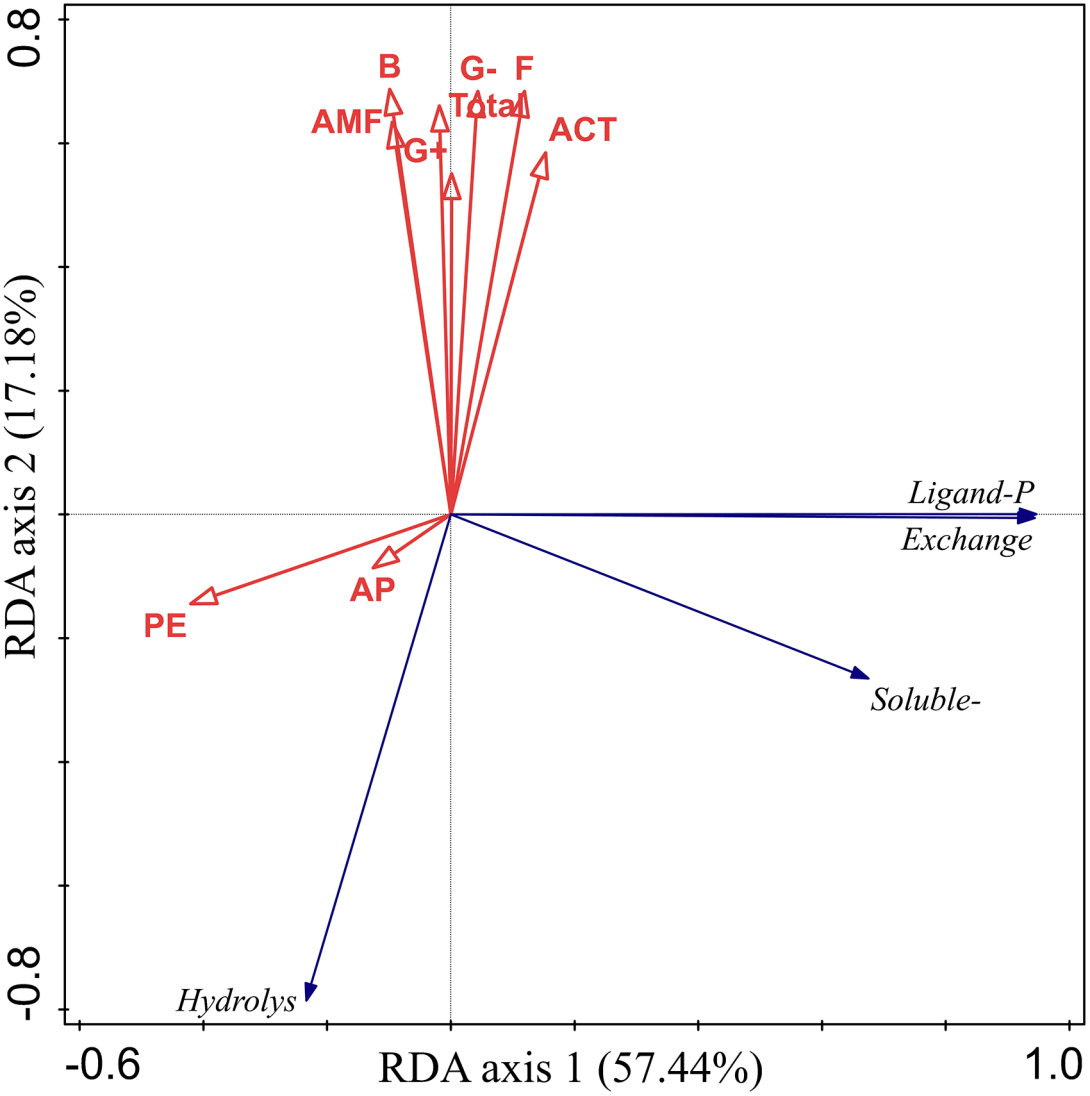
Relationships between soil bioavailable P fractions and AP, PE, and microbial community composition as examined by redundancy analysis. AP, acid phosphatase; PE, phytase; F, fungi; ACT, *Actinomyces*; AMF, arbuscular mycorrhizal fungi; G−, Gram−; G+, Gram+; B, bacterial.

The RDA results for bioavailable P, PE, AP, and microbial community composition in the rhizosphere soil showed that soil biochemical properties explained 74.62% of the variation in soil P fractions. The first axis explained 57.44% of the total variation, and the second axis explained 17.18% of the total variation (Figure 7). In addition, hydrolyzable P was positively correlated with AP but negatively correlated with microbial community composition. Soluble P, exchangeable P, and ligand P had high positive correlations, and all of them had a high negative correlation with PE.

## Discussion

### Effects of stand age on the TP in the rhizosphere soil

The results of this study showed that the TP concentration in the rhizosphere soil decreased first, and then increased with an increase in stand age. The change pattern can be explained based on the process of P cycling in the ecosystem, that is, at the initial period stages after *P. massoniana* are planted, trees absorb P from the rhizosphere soil for growth and development, resulting in the consumption of P in the rhizosphere soil (Turner and Lambert, 2010). Therefore, when the *P. massoniana* plantation was less than 19 years, the TP concentration in the rhizosphere soil decreased continuously. With increasing stand age, the input of litter increased, and the decomposition process continued, such that P in the litter was returned to the soil (Chen et al., 2020). Therefore, when the *P. massoniana* plantation was older than 9 years, the concentration of TP in the rhizosphere soil increased continuously. It is worth noting that the rhizosphere soil TP concentration at 35 years was only 66.04% of that at 5 years, indicating that the return of P in the *P. massoniana* plantation is very slow. Data from several studies suggest changes in litter yields in plantations compared to that of natural forests (Yang et al., 2009; Maranguit et al., 2017). For instance, the annual litter yield of a natural broad-leaved forest was 13.39 t ha^−1^ y^−1^, while that of a *P. massoniana* plantation was only 7.61 t ha^−1^ y^−1^, and less litter yield has been observed to lead to decreased soil P input (Guo et al., 2016). Previous research has established that it takes 27 years for soil carbon concentration to recover to the level of an evergreen broad-leaved forest after plantation, which is much shorter than the recovery time of P concentration (Zhang et al., 2013). The N:P ratio can be used as a diagnostic index of P supply and can be used to determine the threshold of nutrient limitation, indicating the supply of soil nutrients during plant growth (Bui and Henderson, 2013; Zhao et al., 2015). In this study, the N:P ratio first increased, and then decreased with increasing stand age, reaching a maximum at 19 years. This is also because in the early stages, *P. massoniana* plantations have a strong demand for P, and with the increase in stand age, the increase in litter input alleviates the P limit. Therefore, attention should be paid to soil P management in the management process of *P. massoniana* plantations, especially for timber stands, as the rotation period should be appropriately extended or soil P should be artificially supplemented to increase economic benefits and improve ecosystem services.

### Effect of stand age on the bioavailable P in the rhizosphere soil

It is well known that the bioavailability of P in soil depends largely on the transformation that takes place between different soil P fractions (Deluca et al., 2015; Fan et al., 2019). Because some soil P fractions are not directly available to plants, transitions between biological P fractions play a key role in determining plant P availability (Richardson et al., 2011; Yang and Post, 2011). In this study, the soluble P concentration changed only to a small extent with the increase in stand age (range, 0.68–0.84 mg P kg^−1^), and the concentration of exchangeable P and ligand P decreased sharply at 9 years (decreased by 88.59% and 81.79%, respectively) and then tended to be constant. In the early stage, the exchangeable P and ligand P decreased because of fine root absorption and consumption, while in the middle and late stages, the content tended to be constant because the input in litter was supplemented (Ma et al., 2007). A ^32^P isotope labeling experiment showed that 99% of P in *Pinus pinaster* seedlings originated from litter, indicating that litter is important for soil P pool replenishment (Jonard et al., 2009). A recent study showed that there is an unambiguous relationship between average annual P uptake and exchangeable P (Wu et al., 2019a,b).

**Figure 8:**
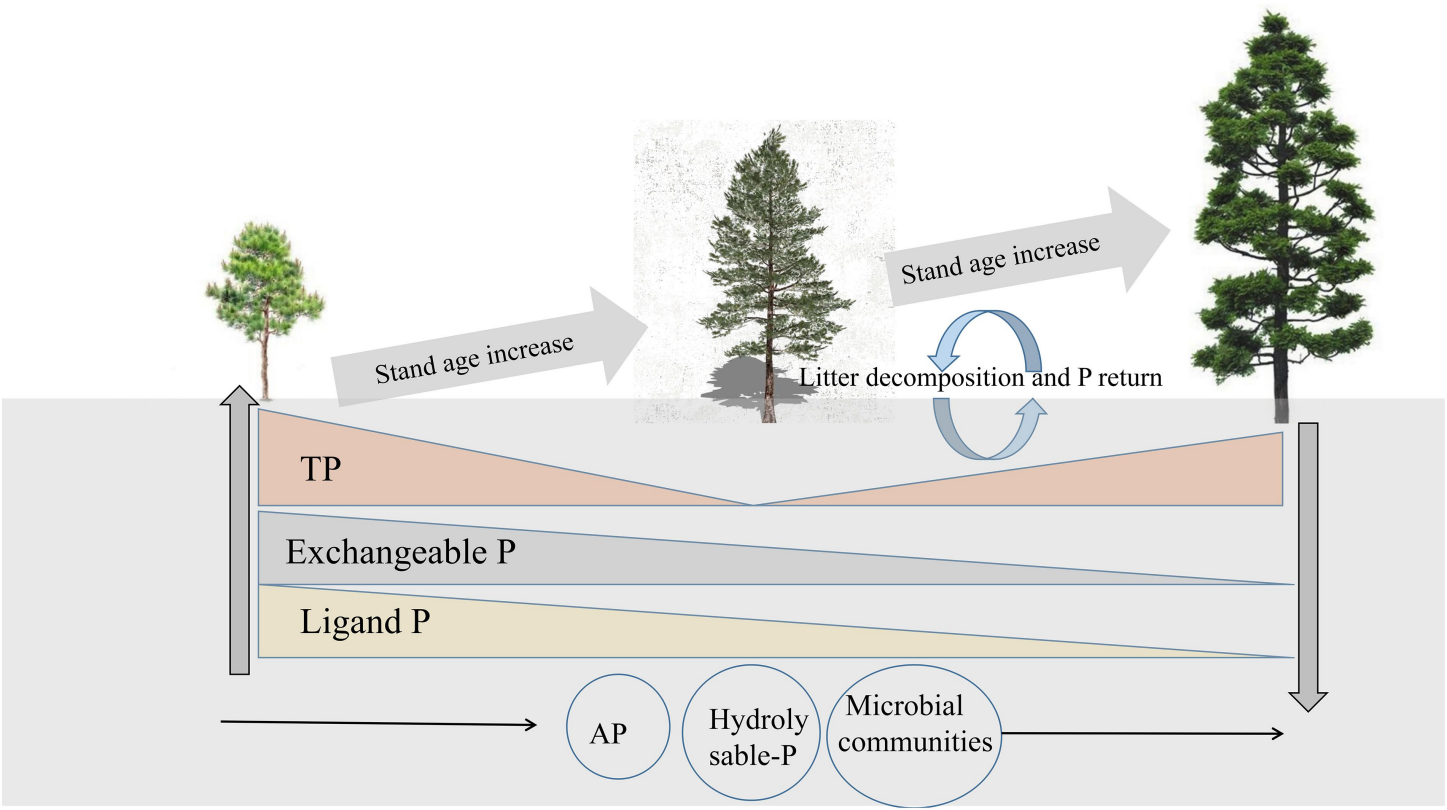
Schematic representation of the changes in rhizosphere soil bioavailable P and total P (TP) in relation to the changes in stand age.

We found that the change in ligand P was strongly correlated with exchangeable P, and exchangeable P was the main reason for the change in soluble P concentration. This is because there is a dynamic balance between exchangeable, ligand, and soluble P. Several lines of evidence suggest that plant roots can secrete citric acid, which can promote the transformation of exchangeable P to soluble P, and the concentration of citric acid decreases with increasing stand age (Fan et al., 2019; Wu et al., 2019a,b). This explains why exchangeable P causes variation in soluble P concentration, and it also provides support for the research result that exchangeable P concentration tends to be constant in the middle and late periods. In addition, we found that hydrolyzable P first increased and then decreased and was positively correlated with AP but negatively correlated with the microbial community composition. The relationships between soil microbial biomass, alkaline phosphatase activity, and P fractions could be explained based on several mechanisms. First, soil microbial activity can accelerate soil Po mineralization rate, and thus mobilize more Pi from both insoluble and Po sources (Richardson and Simpson, 2011). Second, some P soluble soil microorganisms can significantly promote the acidification of alkaline soil through various mechanisms, such as promotion of the production and secretion of organic acids (Eisenhauer et al., 2017). In addition, several studies have reported that increased microbial biomass and diversity generally promote the activity of related phospho-soluble soil enzymes, such as acid phosphatase (Kim et al., 1998), ultimately leading to the transfer of more P to the unstable P pool, which is also consistent with the results of our SEM. Therefore, the change in hydrolyzable P may be due to the comprehensive action of various P fractions, and concentration of hydrolyzable P is positively and significantly correlated with AP and negatively and significantly correlated with the microbial community (Figure 8).

## Conclusion

Stand age has strong effects on P availability, as reflected by the composition of available P fractions in the rhizosphere soil. In general, in the early stages of *P. massoniana* plantations, P demand was high and organic acids were secreted. Exchangeable P and ligand P were converted into soluble P for plant absorption and utilization, resulting in a decrease in TP concentration in the rhizosphere soil with increasing stand age. In the middle and late stages, the secretion of citric acid decreased, the consumption of exchangeable P and ligand P in the soil decreased, and the concentrations of exchangeable P and ligand P in the soil tended to remain constant. At the same time, the nutrient supplement in the litter gradually increased, and P in the plant gradually returned to the soil, resulting in an increase in the TP concentration in the rhizosphere soil with increasing stand age. Meanwhile, soil microbial community composition and AP-driven hydrolyzable P changes played an important intermediate role in all the stand ages. In short, these results reflect the potential relationship between different bioavailable P pools in rhizosphere soils, which could help to deepen the understanding of soil P cycle during the development of *P. massoniana* plantations and provide a reference for artificial intervention.

## Data availability statement

The raw data supporting the conclusions of this article will be made available by the authors, without undue reservation.

## Author contributions

XG and BZ contributed to the conception of the study. YX contributed significantly to analysis and manuscript preparation, performed the data analyses, and wrote the manuscript. WX and LL helped to perform the analysis with constructive discussions. All authors contributed to the article and approved the submitted version.

## Funding

This study was supported by the Fundamental Research Funds for the Central Non-profit Research Institution (CAFYBB2021QD002 and CAFYBB2020ZE001) and Qianjiangyuan National Forest Ecological Research Station.

## Conflict of interest

The authors declare that the research was conducted in the absence of any commercial or financial relationships that could be construed as a potential conflict of interest.

The reviewer ZJ declared a shared affiliation with the authors at the time of the review.

## Publisher’s note

All claims expressed in this article are solely those of the authors and do not necessarily represent those of their affiliated organizations, or those of the publisher, the editors and the reviewers. Any product that may be evaluated in this article, or claim that may be made by its manufacturer, is not guaranteed or endorsed by the publisher.
